# Clear Victory for *Chlamydia*: The Subversion of Host Innate Immunity

**DOI:** 10.3389/fmicb.2019.01412

**Published:** 2019-07-03

**Authors:** Hongliang Chen, Yating Wen, Zhongyu Li

**Affiliations:** ^1^ Institute of Pathogenic Biology, Hengyang Medical College, Hunan Provincial Key Laboratory for Special Pathogens Prevention and Control, Hunan Province Cooperative Innovation Center for Molecular Target New Drug Study, University of South China, Hengyang, China; ^2^ Department of Clinical Microbiology Laboratory, Chenzhou No. 1 People’s Hospital, Chenzhou, China

**Keywords:** *Chlamydia*, innate immune response, immune recognition, innate immune cells, survival and growth

## Abstract

As obligate intracellular bacterial pathogens, members of the *Chlamydia* genera are the pivotal triggers for a wide range of infections, which can lead to blinding trachoma, pelvic inflammation, and respiratory diseases. Because of their restricted parasitism inside eukaryotic cells, the pathogens have to develop multiple strategies for adaptation with the hostile intracellular environment—intrinsically present in all host cells—to survive. The strategies that are brought into play at different stages of chlamydial development mainly involve interfering with diverse innate immune responses, such as innate immune recognition, inflammation, apoptosis, autophagy, as well as the manipulation of innate immune cells to serve as potential niches for chlamydial replication. This review will focus on the innate immune responses against chlamydial infection, highlighting the underlying molecular mechanisms used by the *Chlamydia* spp. to counteract host innate immune defenses. Insights into these subtle pathogenic mechanisms not only provide a rationale for the augmentation of immune responses against chlamydial infection but also open avenues for further investigation of the molecular mechanisms driving the survival of these clinically important pathogens in host innate immunity.

## Introduction Of *Chlamydia*

**Figure 1:**
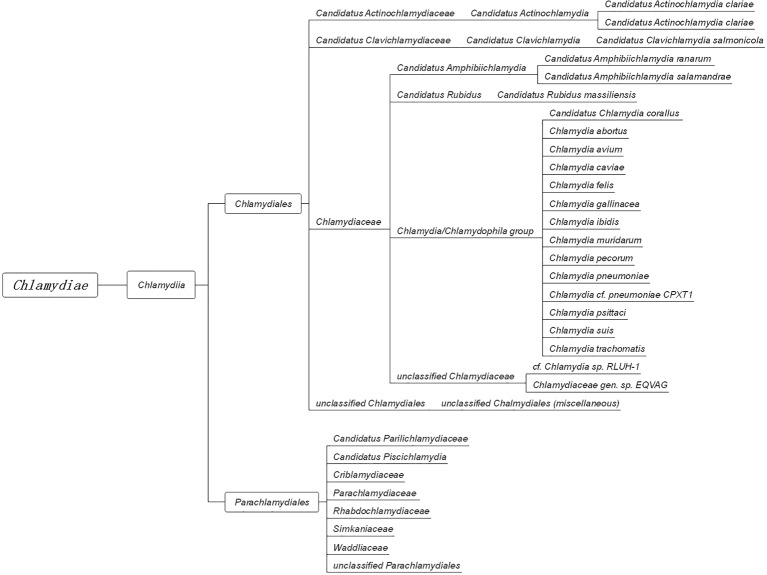
The taxonomy of Chlamydia. The phylum Chlamydiae comprises a single class, Chlamydia, containing two orders, Chlamydiales and Parachlamydiales. The order Chlamydiales encompasses four families, Candidatus Actinochlamydiaceae, Candidatus Clavichlamydiaceae, Chlamydiaceae, and the unclassified Chlamydiales. The family Chlamydiaceae contains the best-known human and animal chlamydial pathogens, such as *C. trachomatis, C. pneumoniae, C. psittaci*, and *C. muridarum*. The other order, Parachlamydiales, encompasses six families.

In 1907, Halberstaedter and von Prowazek observed oval cytoplasmic inclusion bodies near the nuclei of conjunctival epithelial cells, derived from the conjunctival scrapings of an experimentally infected orangutan (Halberstädter and Prowazek, 1907). The inclusion bodies, which were first mistakenly considered to be protozoa and later viruses, were named Chlamydozoa (after the Greek word *chlamys*, meaning cloak) and described as the causal agents of trachoma. This purported *Chlamydia*-like organism was first isolated from patients with trachoma by Tang et al. (1956). Since then, interests in *Chlamydia* research has rocketed, accompanied by significant progress in the areas of basic microbiology, pathogenesis, and immunology. With the discovery and the extensive characterization of new species of *Chlamydia* by novel genetic tools and complete genome sequencing, an emendation of phylum Chlamydiae was proposed (Harris et al., 2012; Kokes et al., 2015; Sixt and Valdivia, 2016). The phylum Chlamydiae (Figure 1) currently consists of a single class (Chlamydia) comprising two orders (Chlamydiales and Parachlamydiales) and nine families. The Chlamydiaceae family originally consisted of the *Chlamydophila* and *Chlamydia* genera, which comprised eight validly named genera and 14 Candidatus genera.

The Chlamydiaceae family encompasses multiple species, all characterized by their ability to propagate within eukaryotic cells, thus acting as infectious agents in many important human and animal diseases (Corsaro and Greub, 2006; Elwell et al., 2016). *Chlamydia trachomatis* and *Chlamydia pneumoniae* are the main species responsible for a wide range of diseases in humans. It has been reported that *C. trachomatis* causes blinding trachoma (serovars A–C), urogenital tract infections (serovars D–K), and systemic lymphogranuloma venereum (LGV) disease (serovars L1–L3). Meanwhile, *C. pneumoniae* is mainly responsible for inciting pneumonia, pharyngitis, bronchitis, and atypical pneumonia. Pathogenic chlamydial isolates, such as *Chlamydia suis, Chlamydia muridarum, Chlamydia caviae*, and *Chlamydia felis*, have been characterized in a variety of animal hosts, including pigs, mice, guinea pigs, and cats. In addition, chicken, cattle, and sheep have been identified as the natural hosts for *Chlamydia psittaci, Chlamydia pecorum*, and *Chlamydia abortus* infections, respectively (Zhong, 2017).

All *Chlamydia* spp. commonly display a unique biphasic developmental cycle alternating between two morphologically distinct forms known as the elementary body (EB) and the reticulate body (RB) (Abdelrahman and Belland, 2005; Grieshaber et al., 2018). Extracellular EBs are capable of invading susceptible cells and, upon entry, differentiating into RBs. The RBs then undergo binary fission, which leads to their asynchronous differentiation back into EBs. Therefore, the *Chlamydia* spp. produce several infectious progenies after only a few rounds of replication, causing rapid progression of infection. This infection process activates the host’s innate and adaptive immune responses by producing multiple cytokines and chemokines, as well as recruiting immune cells such as polymorphonuclear and mononuclear leukocytes, T cells, and B cells (Beagley et al., 2009; Moore-Connors et al., 2013; Rajeeve et al., 2018). Despite the strong, long-lasting immune response mounted by the host, the *Chlamydia* spp. are still considered to dominate the battlefield. Due to their many defenses, evolved to manipulate host immune responses and prevent pathogen clearance, some clinical chlamydial infections persist asymptomatically for months (Hanada et al., 2003; Geisler, 2010; Gottlieb et al., 2010).

Innate immunity acts as the first line of defense against invading *Chlamydia*, by triggering an inflammatory response and empowering the highly specialized adaptive immune system to confer long-lasting immunological memory. Thus, the key to maintain chlamydial intracellular survival and persistence is the circumvention of the host innate immune response. This review will mainly focus on the cross-talk that exists between *Chlamydia* and host innate immunity, based on recent research and our own work in the field.

## Innate Immune Recognition Of *Chlamydia*

### Innate Immunity

The innate immune system represents an ancient evolutionary defense strategy acting as a physical and chemical barrier against infectious agents at a molecular and cellular level, and thus priming the highly specialized adaptive immune response. As a component of the innate immune system, mucosal barriers are the first line of defense against pathogenic invasion. These barriers are formed by epithelial cells, as well as the substances they secrete (France and Turner, 2017). If any of the mucosal barrier components are compromised, the host’s mucosal defenses will be breached. For example, the administration of levonorgestrel or depot-medroxyprogesterone acetate (DMPA) to mice increased genital mucosal permeability, causing these animals to become more susceptible to *Chlamydia* infection and persistence (Vicetti Miguel et al., 2018). As is the case for most bacterial infections, the transmigration of *Chlamydia*-infected cells through the mucosal barrier grants the pathogen access to the lymphatic system. Consequently, the pathogen is able to trigger a variety of host innate immune responses (Bulut et al., 2002; Erridge et al., 2004; Zou et al., 2016), such as the activation of pattern recognition receptors (PRRs) on the surface of innate immune cells. PRRs recognize highly conserved pathogen-associated molecular patterns (PAMPs) and include Toll-like receptors (TLRs), C-type lectin receptors (CLRs), and scavenger receptors. Cytoplasmic PRRs, such as nucleotide-binding oligomerization domain (NOD)-like receptors and RIG-I-like receptors (RLRs), are able to sense intracellular bacteria (Moresco et al., 2011). On the contrary, cytoplasmic PRRs such as NOD-like receptors and RLRs are able to sense intracellular bacteria. Owing to their distinctive developmental cycle, the *Chlamydia* spp. are often regarded as facultative intracellular organisms. The EBs of *Chlamydia* spp. can be released outside of the host cell by mature RBs, enabling the recognition of *Chlamydia* by both intracellular and extracellular PRRs. As the PRRs are distributed across the surface or within the cytoplasm of over 20 types of innate (comprising monocytes, neutrophils, macrophages, and dendritic cells) and adaptive (comprising T and B lymphocytes) immune cells, as well as epithelial cells, it is very difficult for *Chlamydia* to evade recognition by the PRRs.

### Innate Immune Recognition of *Chlamydia* by Toll-Like Receptors

**Figure 2:**
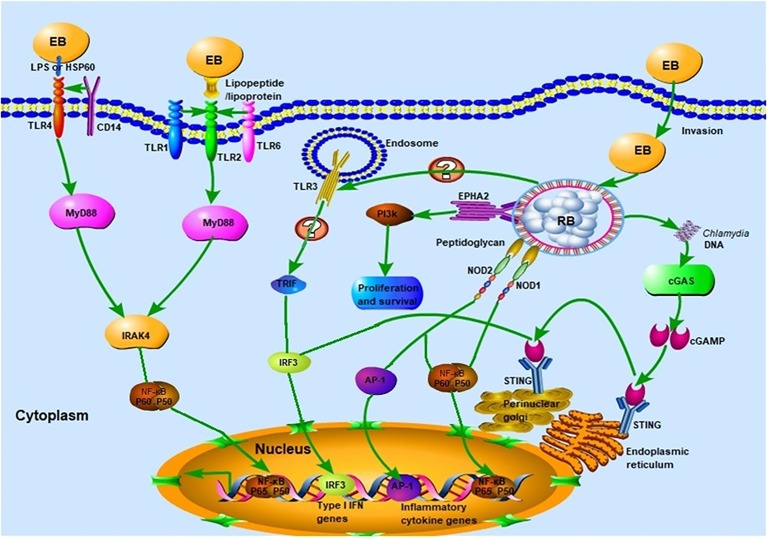
Innate immune recognition of *Chlamydia.* Chlamydial infection can be recognized by pattern recognition receptors (PRRs) such as Toll-like receptors (TLRs), NLRs, and cGAS, which elicit strong host innate immune responses and the release of several pro-inflammatory cytokines. TLR1/2 and TLR2/6 recognize chlamydial lipopeptide/lipoprotein, and TLR4 recognizes chlamydial LPS and HSP60. Upon binding of cognate ligands, the MyD88-dependent pathway, which is common to all TLR signaling (with the exception of TLR3) is immediately activated. Whether TLR3 is involved in the recognition of chlamydial infection remains to be elucidated. The chlamydial peptidoglycan binds to the NLRs and induces the production of pro-inflammatory cytokines *via* nuclear factor-κB (NF-κB) or activator protein 1 (AP-1) signaling. The stimulation of cGAS by *Chlamydia* spp. DNA leads to the dimerization and activation of IRF3, which then translocates into the nucleus and promotes the transcription of type I IFN and IFN-inducible genes. Chlamydial components also bind to the ephrin receptor A2 (EPHA2), which in turn triggers the activation of PI3K-downstream signaling, favoring the proliferation and survival of *Chlamydia*.

The TLRs, of which there are 10 in humans and 13 in mice, are vital participants in the initiation of the innate immune response against microbial invaders. They achieved their function by collectively recognizing the lipid, carbohydrate, peptide, and nucleic acid components that make up pathogens (Moresco et al., 2011). Chlamydial components recognized by TLRs (Figure 2) include chlamydial lipopolysaccharide (LPS, bound by TLR2), cHSP60 (bound by TLRs 2 and 4), and lipopeptide/lipoprotein (bound by TLRs1/2 or TLRs2/6) (Bulut et al., 2002; Erridge et al., 2004; Bas et al., 2008). There is also speculation that a novel, as yet uncharacterized, ligand binds to and signals through TLR3 during *Chlamydia*-induced genital tract infection (Derbigny et al., 2010; Carrasco et al., 2018). It is worth noting that biochemical analysis has revealed that both the EBs and RBs lack peptidoglycan (PGN) (Chopra et al., 1998). However, a functional PGN pathway reportedly exists in *Chlamydia*, and the *Chlamydia* spp. are sensitive to penicillin, a PGN synthesis inhibitor. Moreover, the full complement of genes required for PGN biosynthesis and assembly are present in chlamydial genomes, which further disproves the “glycanless peptidoglycan” hypothesis (Liechti et al., 2014; Packiam et al., 2015). The reasons for the above paradox may be attributed to (1) the low quantity of PGN within the chlamydial cell wall, which could not be detected by the methodologies available a decade ago; or (2) the possibility that *Chlamydia* may instead produce a yet uncharacterized glycanless cell wall polypeptide similar to PGN. Several studies have demonstrated the role of TLR2 (receptor for PGN) and its adaptor, myeloid differentiation primary response protein 88 (MYD88), in bacterial recognition. This receptor may respond to *Chlamydia* infection by virtue of its localization around the periphery of the chlamydial inclusion, followed by intracellular signal transmission. The TLR4-mediated recognition of chlamydial LPS and HSP60 (cLPS and cHSP60, respectively) mediates dendritic cell (DC) maturation and the release of cytokines during *C. pneumoniae* infection (Bulut et al., 2002). In addition, LPS isolated from *C. trachomatis* (strain LGV-1) has been reported to induce TLR2-mediated nuclear factor-κB (NF-κB) activation too (Erridge et al., 2004). CD14, a PRR on monocytes and macrophages, also functions as a signaling receptor for bacterial LPS (Kol et al., 2000). This high-affinity receptor was involved in the macrophage infectivity potentiator-mediated pro-inflammatory cytokine response to the *C. trachomatis* EBs within human macrophages. The polymorphism of CD14 was also reportedly associated with *Chlamydia*-stimulated tumor necrosis factor alpha (TNF-α) production (Eng et al., 2004; Bas et al., 2008). To date, there has been little consensus on which of the aforementioned TLRs is the predominant receptor required for the effective recognition of *Chlamydia* during infection.

### Immune Recognition of *Chlamydia* by Other Pattern Recognition Receptors

Besides the TLRs, other PRRs, which include components such as NOD, cyclic GMP-AMP synthase (cGAS), and STING, can also recognize chlamydial PAMPs (Figure 1; Kavathas et al., 2013). Several studies have reported that live intact *Chlamydia* particles are required for PRR activation. For instance, dead *C. pneumoniae* failed to activate either NOD1 or NOD2 PRRs in HEK293 cells (Opitz et al., 2005). Furthermore, live but not UV-inactivated *C. muridarum* induced TLR2-dependent cytokine expression (Darville et al., 2003; Rank et al., 2010). In addition to PRRs, other receptors are involved in chlamydial recognition. The cell surface tyrosine kinase Ephrin A2 receptor (EphA2), known as a chlamydial invasion receptor, was shown to bind to *C. trachomatis*, activate PI3 kinase (PI3K) (Subbarayal et al., 2015), and promote chlamydial replication.

The inflammasome is also able to sense cellular stress signals caused by chlamydial infection. In addition, components such as pORF5, encoded by the chlamydial plasmid, are essential for the initiation of effective host defenses against microbes (Webster and Goodall, 2017). Collectively, the TLRS and PRRs are activated to varying degrees depending on the bacterial species and the host cell types involved in the recognition of foreign antigen. The impact of such factors on chlamydial survival and bacterial clearance during infection will be discussed at a later stage in the review.

## Subversion Of The Host Innate Immune Response By *Chlamydia*

### Interference With Nuclear Factor-κB Signaling

The NF-κB protein complex serves as a prominent inducible transcription factor, which is present in the majority of animal cell types and is responsible for regulating gene transcription as part of the innate immune response. The NF-κB transcription factor is often located in an inactive state in the cytosol, where it associates with the inhibitory protein IκBα. Extracellular signals relating to pathogen encounter lead to the activation of the enzyme IκB kinase (IKK), resulting in the phosphorylation and subsequent dissociation of IκBα. Once IκBα is released from NF-κB, it is targeted for degradation by the proteasome (Rothschild et al., 2018). Meanwhile, the activated NF-κB translocates to the nucleus to assist with the transcription of specific genes.

The *Chlamydia* spp. use various strategies to interfere with the function of NF-κB, including (1) blocking the degradation of the NF-κB retention factor, IκBα and (2) preventing the nuclear translocation of NF-κB, thus stopping or dampening NF-κB transcription. *C. trachomatis* encodes two proteins with deubiquitinating (DUB) activity, ChlaDub1 and ChlaDub2 (Le Negrate et al., 2008). ChlaDub1 (also known as CT868) binds to the NF-κB inhibitory subunit I𝜅Ba, together with two covalently bound cyano-pyrimidines, as well as with its substrate ubiquitin. The formation of this complex inhibits the ubiquitination and degradation of I𝜅Bα and stabilizes it in the cytosol. The ectopic expression of ChlaDub1 also blocks NF-κB signaling downstream of the IKK complex but does not interfere with the upstream components of the pathway, leading to the suppression of NF-κB activation (Le Negrate et al., 2008; Ramirez et al., 2018). Once in the host cytoplasm, the *C. pneumoniae*-specific inclusion membrane protein (Inc) CP0236 binds to and alters the distribution of NF-κB activator 1 (Act1) in the cytoplasm. This sequestration of Act1 suppresses CP0236 recruitment to the interleukin-17 (IL-17) receptor and enables *C. pneumoniae* to inhibit NF-κB activation triggered by IL-17, in IL-17-stimulated cells (Wolf et al., 2009). In the fallopian tube model, *C. trachomatis* suppresses NF-κB activation by inducing the production of the stem cell marker olfactomedin 4 (OLFM4), through the Wnt-dependent signaling pathway (Kessler et al., 2012; Kintner et al., 2017). Additionally, the tail-specific protease of *C. trachomatis* (CT441) and the chlamydial protease-like activity factor (CPAF) of *C. pneumoniae* are involved in suppressing NF-κB signaling. CPAF cleaves the p65/RelA component of the NF-κB pathway, effectively suppressing the immune response in *Chlamydia*-infected cells (Lad et al., 2007; Christian et al., 2010). In summary, the impairment of NF-κB activation appears to be a major mechanism utilized by *Chlamydia* to weaken the host’s immune response and facilitate the long-term survival of this opportunistic pathogen.

### Interference With Interferon Signaling

Interferon (IFN) signaling is an important element of the immune system that contributes to the eradication of multiple pathogens, including viruses, bacteria, and intracellular parasites. IFN gamma (IFN-γ), the only member of the type II class of IFNs, is an essential component of IFN signaling. IFN-γ is produced predominantly by natural killer (NK) cells, natural killer T (NKT) cells, cytotoxic CD8^+^ T lymphocytes, and non-cytotoxic innate lymphoid cells, upon adequate stimulation (Okamura et al., 1998; Thale and Kiderlen, 2005). NK cells are thought to be the main source of innate IFN-γ (Thale and Kiderlen, 2005), with DCs also allegedly being capable of producing the cytokine under certain conditions (Frucht et al., 2001). The *Chlamydia* spp. are among the first non-viral pathogens to have been reported to induce IFN-γ production, leading to alterations in intracellular chlamydial growth in *in vitro* cell culture systems (Thomas et al., 1993; Elwell et al., 2016; Ziklo et al., 2016).

IFN-γ typically restricts chlamydial growth through a tryptophan depletion or a p47 GTPase interference mechanism in human or murine epithelial cells, respectively (Nelson et al., 2005). In humans, IFN-γ induces the production of the tryptophan-decyclizing enzyme indoleamine 2,3-dioxygenase (IDO), a non-constitutive enzyme, which catalyzes the degradation of tryptophan to kynurenine and *N*-formylkynurenine, thereby starving *Chlamydia* of this essential amino acid. The IDO-mediated exogenous depletion of tryptophan prevents the microorganism from differentiating into infectious EBs, in addition to inhibiting the replication of any tryptophan auxotrophic *Chlamydia* strains. *Chlamydia* serovars and species display a range of susceptibilities to the inhibitory effects of IFN-γ treatment *in vitro* (Caldwell et al., 2003). For instance, the IFN-γ-mediated growth inhibition of *C. trachomatis* varies depending on the human cell line used in the experiment (Hela, A549, ME180, HEp-2, and A2EN cells have all been tested), which may be due to host cell-intrinsic differences (Sherchand et al., 2016). This effect, however, is yet to be observed in murine systems, despite the presence of 2,3-IDO gene in mice in other situations. Human, but not mouse chlamydial strains, avoid this response by impairing the production of IFN or counteracting its downstream gene products in order to persist in eukaryotic cells.

Similarly, *C. pneumoniae* expresses a unique protease, which it employs to degrade the signaling molecule TNF receptor-associated factor 3 (TRAF3). TRAF3, in turn, blocks the phosphorylation of IFN regulatory factor 3 (IRF3) and the subsequent induction of IFN-β (Wolf and Fields, 2013). Meanwhile, *C. trachomatis* inhibits the production of IFN through TepP (also known as CT875), which is able to downregulate the expression of IFN-induced protein with tetratricopeptide repeats 1 (IFIT1) and IFIT2 (Chen et al., 2014). *C. trachomatis* genital serovars express a functional tryptophan synthase enzyme (trpBA), which enables these serovars to circumvent the shortage of tryptophan by using indole molecules provided by the local microbiota as a substrate for tryptophan synthesis (Ziklo et al., 2016). By contrast, the *C. trachomatis* ocular serovar is more sensitive to an IFN-γ-rich, tryptophan-limiting environment as a result of its nonfunctional tryptophan synthase. *C. pecorum* also expresses *trp* genes, which render it completely resistant to IFN-γ-mediated tryptophan depletion (Caldwell et al., 2003). Instead, it has been shown to scavenge tryptophan in bovine kidney epithelial cells, demonstrating that bovine IFN-γ is unable to suppress the growth of *C. pecorum.* The growth of *C. trachomatis* is (Islam et al., 2018), however, inhibited in the same setting, further proving that the relationship between IFN-γ and *Chlamydia* is host-specific in nature. In murine epithelial cells, IFN-γ induces the expression of p47 GTPases, which are potent inhibitors of chlamydial growth. As seen in humans, the murine *Chlamydia* strains have coevolved with their host and acquired the ability to produce a large toxin possessing YopT homology, to circumvent host GTPases.

Another possible mechanism by which IFN-γ could inhibit chlamydial growth depends on NO production by the IFN-γ inducible NO synthase (iNOS) (Ramsey et al., 2001; Abu-Lubad et al., 2014), which possesses antimicrobial activity by causing DNA damage, protein nitration, and lipid peroxidation. However, some *Chlamydia* species have also evolved strategies to bypass these host defenses. For instance, *C. trachomatis* and *C. pneumoniae* can manipulate polyamine and NO synthesis pathways (Caldwell et al., 2003; Abu-Lubad et al., 2014) by promoting ornithine decarboxylase (ODC) expression and reducing iNOS levels. Hypoxia was also reported to reduce the anti-microbial activity of IFN-γ in the context of persistent *C. trachomatis* infection, indicating that the anti-chlamydial activity of IFN-γ is reduced in the low-oxygen environment that is typical of genital *C. trachomatis* infections (Jerchel et al., 2014). However, the precise molecular mechanisms remain unknown.

### Interference With Inflammation

*Chlamydia* researchers worldwide hold a general view that inflammation during chlamydial infection is somewhat of a “double-edged sword.” Upon being recognized by the innate immune system, the *Chlamydia* spp. trigger inflammatory responses. This inflammatory process is critical for pathogen clearance, but may also promote pathogen persistence and increase host morbidity in an environment of ongoing inflammation. Although the *Chlamydia* spp. prefer to replicate in non-immune cells, they will also infect immune cells as well as epithelial cells (Herweg and Rudel, 2016). *Chlamydia*-infected host cells produce a number of cytokines and chemokines, including CXC-chemokine ligand 1 (CXCL1), CXCL8 (also known as interleukin-8, IL-8), TNF-α, and IL-1β *via* various signaling pathways (Perfettini et al., 2003b). These pro-inflammatory mediators recruit immune cells to the site of infection and cause local inflammation and tissue damage, resulting in the pathology of chlamydial infection.

The combination of cytokines secreted, as well as their expression levels, vary according to the infecting *Chlamydia* species and the host cell type. For example, infected epithelial cells secrete the cytokines IFN-γ, IFN-α, IFN-β, and IL-12. Meanwhile, infected monocytes produce IL-4 and IL-10. Notably, different concentrations of IL-8 and IL-6 are detected at the same infectivity ratio in infected cervical HeLa cells (Sherchand et al., 2016; Sixt and Valdivia, 2016; Du et al., 2018). Infection of epithelial cells and macrophages with *Chlamydia* can also trigger inflammasome activation (including the NLRP3/ASC inflammasome), which is a tightly regulated process designed to prevent the excess accumulation of inflammatory mediators. However, a study of *C. trachomatis* indicates that the chlamydial protein CpoS can inhibit host inflammasome responses (Sixt et al., 2017). Additionally, the activation of the NLRP3/ASC inflammasome also requires the generation of reactive oxygen species (ROS), lysosomal damage, and cytosolic K^+^ efflux. Once activated, the NLRP3/ASC inflammasome mediates caspase-1-dependent IL-1β signaling activation that regulates the maturation of IL-1β and IL-18 (Webster and Goodall, 2017), and interferes with chlamydial infectivity, thus defending the host against chlamydial infection.

Although the inflammatory response is necessary for the immune-mediated clearance of *Chlamydia*, the long-term damage caused by chronic inflammation is often observed in trachoma and other chlamydial diseases. The *Chlamydia* spp. carry an arsenal of weapons to orchestrate the innate immune response by either inhibiting or enhancing the production of pro-inflammatory cytokines, promoting chlamydial persistence under different circumstances. A good case in point is IL-10, which is recognized as an anti-inflammatory cytokine, and plays a key role in the suppression of immune responses by inhibiting several pro-inflammatory molecules in the immune response against chlamydial infection. Moniz and colleagues identified that both *C. trachomatis* and *C. muridarum* induced IL-10 production in infected macrophages and plasmacytoid DCs (Moniz et al., 2009; Azenabor and York, 2010). Similar to the findings from *in vitro* studies, the increased expression of IL-10 in the semen and serum of patients infected with *C. trachomatis* was also determined (Hakimi et al., 2014). Besides, chlamydial CPAF was able to cleave p65/RelA, a transcription factor required for NF-κB signaling, leading to a reduction in the IL-1β-dependent secretion of IL-8, in human and murine cells (Christian et al., 2010; Jorgensen et al., 2011). Hence, the secretion of CPAF represents a hypothetical mechanism that acts to reduce host cell sensitivity to a pro-inflammatory stimulus, which may contribute to bacterial growth *in vivo*.

LPS commonly presents on Gram-negative bacilli and is capable of eliciting inflammatory cytokines and stimulating phagocytic cells (Ingalls et al., 1995). *C. trachomatis* LPS is a major chlamydial surface antigen. It is, however, significantly (~100-fold) less potent at activating host immune cells than *Salmonella* LPS or gonococcal LOS, revealing only a minor role for *C. trachomatis* LPS in eliciting the pro-inflammatory cytokine response. *Chlamydia* CPAF has also been demonstrated to neutralize the human serum anti-chlamydial activity by cleaving the complement factors B and C3, thus blocking complement activation and attenuating the production of pro-inflammatory cytokines (Yang et al., 2016). Furthermore, microRNA (miRNA)-155, which is reportedly upregulated in chlamydial follicular trachoma infection and correlates with the severity of inflammation, is capable of negatively regulating inflammation by targeting MYD88, a key inflammatory pathway adaptor molecule (Tang et al., 2010; Derrick et al., 2016). This provides a potential mechanism by which *Chlamydia* handles the inflammatory response through the regulation of miRNAs.

### Interference With Apoptosis

Apoptosis can be initiated either when a ligand engages a cell-surface receptor or *via* intracellular cytopathic signals, which are known as extrinsic and intrinsic signaling pathways, respectively. Discordant views regarding the impact of *Chlamydia* on the apoptotic signaling pathways are likely due to the complexity of apoptotic signaling, the unique biphasic developmental cycle of the pathogen, and the host cells used in the studies (Rahman et al., 2015; Sarkar et al., 2015; Matsuo et al., 2018). The *Chlamydia* spp. regulate apoptosis by exploiting host cell mechanisms. As part of their intracellular survival strategy during the replicative phase, *Chlamydia* initially inhibit apoptosis. However, towards the mid to later stages of replication, the microorganism induces apoptosis to enable the propagation of *Chlamydia*. Under some circumstances, the *Chlamydia* spp. also inhibit apoptosis during persistent growth or in phagocytes, but induce apoptosis in immune cells to aid immune evasion. On the other hand, Barbara and colleagues demonstrated that *C. trachomatis*-infected cells, exposed to pro-apoptotic stimuli, predominantly died. In this study, the anti-apoptotic actions of *Chlamydia* were not sufficient to protect the pathogen’s replicative niche (Sixt et al., 2018). And in all, the *Chlamydia* spp. have developed mechanisms to interfere with pro- and anti-apoptotic signals, as well as to correctly time cell death, in order to guarantee their survival and propagation within host cells.

The *Chlamydia* spp. promote cell viability and inhibit cell death at an early stage in their developmental cycle, which can occur through the inhibition of pro-apoptotic pathways and activation of pro-survival pathways. Numerous mechanisms of blocking apoptosis have been reportedly employed by the *Chlamydia* spp.: (1) the prevention of cytochrome c release from the mitochondria by *Chlamydia*-dependent anti-apoptotic factors; (2) the murine double minute 2 (MDM2)-dependent proteasomal degradation of cellular p53, mediated by the activation of the classical MDM2–p53 interaction axis (Gonzalez et al., 2014); (3) the sequestration of the BCL-2-associated agonist of cell death (BAD) to the inclusion membrane *via* 14-3-3β-binding, and of pro-apoptotic protein kinase Cδ (PKCδ) on the inclusion vacuole through binding to diacylglycerol-enriched membranes away from its conventional target sites (Verbeke et al., 2006; Kokes et al., 2015); and (4) the upregulation of the expression of genes that encode anti-apoptotic inhibitors of apoptosis protein (IAP) homologues, BAG family molecular chaperone regulator 1 (BAG1), and BCL-2 family member MCL-1 (Bastidas et al., 2013; Kun et al., 2013). Greene et al. (2004) compared host cell apoptotic responses to infection using 17 different chlamydial serovars and strains (including A–K, L1, L3, Ba, and *C. muridarum*), all of which exhibited clear anti-apoptotic activity, the extent of which varied between serovars. It has been proposed that CPAF contributes to chlamydial anti-apoptotic activity by degrading the pro-apoptotic BH3-only proteins (Fischer et al., 2004; Pirbhai et al., 2006). However, subsequent studies have shown the CPAF-mediated proteolysis to be an artifact of the enzymatic activity present within cell lysates rather than in intact cells (Snavely et al., 2014). Thus, the role of CPAF in mediating anti-apoptotic activity requires further clarification. Moreover, chlamydial infections also activate pro-survival signaling pathways, such as the phosphoinositide 3-kinase (PI3K), the mitogen-activated protein kinase (MAPK, also known as ERK), the MAPK kinase (MAPKK, also known as MEK), and the Wnt/β-catenin signaling pathways (Verbeke et al., 2006; Elwell et al., 2008; Kessler et al., 2012; Kun et al., 2013), through the interaction of *Chlamydia* with fibroblast growth factor receptor (FGFR) or the receptor tyrosine kinases (RTKs) and the ephrin receptor A2 (EPHA2). The regulation of these pathways by the *Chlamydia* spp. appears to be central to the activation of pro-survival genes and the expression of anti-apoptotic factors within host cells, enabling these bacteria to elicit the long-lasting survival signals required for replication.

The apoptosis of host cells can also be triggered by chlamydial products and host cytokines such as TNF, through the caspase-independent programmed cell death pathway (Miyairi and Byrne, 2006). Several studies have demonstrated that the activation of either of the pro-apoptotic proteins (BAX and BAK) does not involve caspases during the latter stages of chlamydial infection (Perfettini et al., 2003a; Zhong et al., 2006). This ultimately leads to host cell lysis and facilitates the efficient release of reorganized EBs from the host cell, in order to initiate new infections. Using bioinformatics approaches, a key apoptotic agent called the *Chlamydia* protein associated with death domain (CADD) has been identified in chlamydial genomes. CADD, regarded as a novel redox protein toxin (Stenner-Liewen et al., 2002; Schwarzenbacher et al., 2004), is composed of two seven-helix bundles, which are crucial for its biological activity. CADD is capable of binding several DD-containing TNF family receptors and can induce apoptosis in a caspase-dependent way when transiently transfected into various mammalian cell lines (Schwarzenbacher et al., 2004). In parallel, the *Chlamydia* spp. can also infect and initiate apoptosis in immune cells such as macrophages and neutrophils. For example, on *C. trachomatis* infection, macrophages and neighboring T cells become susceptible to apoptosis in a caspase-1- (Chen et al., 2017) and TNF-α-dependent manner (Jendro et al., 2004), respectively. *C. pneumoniae* also inhibits the proliferation of activated T cells *via* the initiation of apoptotic pathways (Olivares-Zavaleta et al., 2011). These abilities may function to prevent bacterial clearance *via* the creation of an immunosuppressive environment, favorable for the intracellular survival of *Chlamydia*.

### Interference With Autophagy

Autophagy is a physiological degradation process that occurs within the lysosomes of most cell types. Its main functions are to maintain cellular homeostasis and selectively remove intracellular bacteria or viruses. The intricacy of the relationship between *Chlamydia* and autophagy is difficult to delineate on account of *Chlamydia*-mediated evasion or induction of autophagy, which varies depending on the *Chlamydia* species and cell line involved. Al-Younes and colleagues investigated whether the *C. trachomatis* serovar L2 interacted with the host autophagic pathway and found that the chlamydial inclusion in epithelial cells could evade fusion with autophagosomes. This was evidenced by the co-localization of monodansylcadaverine (MDC) with the cytoplasm of infected cells not containing chlamydial inclusion (Al-Younes et al., 2004). However, autophagy is markedly induced during the replicative stages of *C. trachomatis* infection in LGV disease. Furthermore, the growth of *C. trachomatis* and *C. pneumoniae* is also reported to be impaired following the addition of autophagy inhibitors including 3-methyladenine (3-MA) and bafilomycin A1 (BafA1) (Al-Younes et al., 2004; Al-Zeer et al., 2009; Ouellette et al., 2011).

In subsequent investigations, guanylate-binding proteins (GBPs) and the immunity-related GTPases (IRGs) such as GBP1, GBP2, Irga6, and Irgd, which are able to induce lysis and infection clearance by autophagy, accumulate in the bacterial inclusions of *C. trachomatis*-infected mouse embryonic fibroblasts (MEFs) upon IFN-γ treatment (Al-Zeer et al., 2009; Haldar et al., 2013). Intriguingly, chlamydial growth is enhanced in autophagy-deficient Atg5^−/−^ or Irga6^−/−^ MEFs by IFN-γ stimulation, compared to that in wild-type MEFs (Al-Zeer et al., 2009; Yasir et al., 2011), indicating a pivotal role for these proteins in the autophagy-mediated resistance to *C. trachomatis* infection. On the contrary, *C. muridarum* is more susceptible to the inclusion ubiquitination in IFN-γ-primed human epithelial cells than *C. trachomatis*, resulting in recruitment of GBPs to the inclusion together with ubiquitin-binding protein p62 (Haldar et al., 2016). Eventually, this ubiquitination causes inclusion rupture and triggers the clearance of *C. muridarum*. The above observations and analysis suggest that the *Chlamydia* spp. can inhibit autophagy, evade lysosomal fusion mechanisms, and initiate autophagy (in order to obtain nutrients such as glycolytic and tricarboxylic substrates) under different circumstances. However, the molecular mechanisms involved in each setting have yet to be elucidated.

## The Role Of Innate Immune Cells In Chlamydial Persistence

### Interaction Between Macrophages and *Chlamydia*

A broad variety of innate immune cells such as macrophages, neutrophils, DCs, and mast cells are responsible for mounting “armed” effectors to identify and kill any foreign invaders with the potential to cause disease. The *Chlamydia* spp. have evolved mechanisms to counteract this cellular attack and even to infect these innate immune cells in order to persist within the host (Bastidas et al., 2013). Several studies have described the association between chlamydial infection and conditions such as reactive arthritis, coronary artery disease, and multiple sclerosis.

Several studies described the association for chlamydial infections with reactive arthritis, coronary artery, and multiple sclerosis (Beagley et al., 2009; Herweg and Rudel, 2016). The researchers hypothesized that macrophages may function as potential carriers for transporting the free *Chlamydia* to the sites of inflammatory disease through the circulation. Subsequent investigations have revealed that several *Chlamydia* spp. were able to infect and survive in both human and murine macrophages and cell lines at a conventional multiplicity of infection (MOI), while most of the *Chlamydia* spp. were toxic to the host cells at higher MOIs (Beagley et al., 2009). Of note, the susceptibility of various macrophage cell lines to infection with *Chlamydia* is related to their varied intrinsic features. This diversity might be extended to other innate immune cells.

The *C. trachomatis* serovar L2 and the mouse pneumonitis (MoPn) strain can productively infect the human acute monocytic leukemia (THP-1) cell line and murine peritoneal cavity macrophages (PerCMs), respectively (Coutinho-Silva et al., 2003; Mpiga and Ravaoarinoro, 2006). Meanwhile, the TW-183, AR-39, and TWAR strains of *C. pneumoniae* are the most likely to target human Mono Mac 6 cells, murine alveolar macrophages, and bone-marrow-derived macrophages (BMDMs), respectively (Beagley et al., 2009). Although it is clear that macrophages are not the optimum host cell targets for *Chlamydia*, owing to their powerful ability to engulf and destroy bacteria, chlamydial persistence following the infection of macrophages can be achieved through (1) the formation of aberrant RBs, to overcome imperfect conditions for growth; (2) the interaction with multiple cytoskeletons, the Golgi, and the endoplasmic reticulum, to acquire sufficient nutrients (Paradkar et al., 2008; Sun et al., 2012; Elwell et al., 2016); (3) the up- or downregulation of inflammatory mediators such as TNF-α, IFN-γ, and ILs, to escape eradication by interfering with apoptotic and autophagic pathways (Jendro et al., 2004; Sherchand et al., 2016; Chen et al., 2017); and (4) the production of adhesion molecules such as the intercellular cell adhesion molecule-1 (ICAM-1), to increase macrophage adherence, thus facilitating the migration of EBs to their preferred sites of replication (Yeung et al., 2017). The persistence of the *Chlamydia* spp. in macrophages may also be explained by the immunological pressure applied by T-cell-mediated immunity, which represents the predominant host defense mechanism against chlamydial infections.

### Interactions Between DCs and *Chlamydia*

Studies using human and mouse DCs reveal that the *Chlamydia* spp. are capable of infecting different types of DCs, which are the most powerful antigen-presenting cells. DCs offer a vital link between innate and adaptive immunity, by priming naive T cells and acting as sentinels of the immune response. Depending on their type, the biological characteristics of DCs may slightly differ, which may partially explain the range of susceptibilities displayed by DCs to chlamydial infections. For example, the mouse-specific strain *C. muridarum* is able to infect BMDCs at an MOI of 1 or 3, which may result in the presence of atypical inclusions in ~10% of BMDCs, as seen by positive anti-major outer membrane protein (MOMP) staining (Jiang et al., 2008). A clinical isolate of *C. pneumoniae* can infect human monocyte-derived DCs (moDCs) *in vitro*, leading to a long-lasting infection, as evidenced by the presence of *C. pneumoniae* DNA and antigen up to 25 days post-infection (Wittkop et al., 2006). *C. trachomatis* enters human moDCs in a heparan-sulfate-independent manner, and is detected within the cells as early as 8 h post-infection (Matyszak et al., 2002).

To further investigate the mechanisms of *Chlamydia* infection of DCs, Jose and colleagues (Rey-Ladino et al., 2007) initially infected BMDCs with *C. muridarum*, prior to infecting HeLa cells with the bacterium isolated from *C. muridarum*-infected BMDCs. They found that *C. muridarum* formed both atypical and typical inclusion, which increased significantly on day 9 after infection. They also observed that the *C. muridarum* inclusions isolated from BMDCs replicated poorly in HeLa cells, and this infection seems not to affect the antigen-presenting capability of *C. muridarum*-infected BMDCs. These findings indicate that DCs promote not only the long-term survival but also the growth of *Chlamydia*. In contrast, another study (Ojcius et al., 1998) observed that *C. psittaci* were internalized *via* macropinocytosis and fused with lysosomal compartments in DC cell line (D2SC/1) a few hours after infection. The contrasting results may be due to the distinct *Chlamydia* species and host cells used in these studies. More interestingly, the infection of DCs with *Chlamydia* would induce the production of the cytokines TNF-α, IL-4, and IL-10 (Kaiko et al., 2008) facilitating Th2 immunity and suppressing Th1 development. This represents a further strategy for supporting chlamydial persistence in both DCs and macrophages.

### Interactions Between Mast Cells, Eosinophils, Neutrophils (and Other Innate Immune Cells), and *Chlamydia*

The infection of mast cells with *Chlamydia* elicits the secretion of cytokines such as TNF-α and IL-4, which promotes the infiltration of immune cells into the airways by opening tight junctions, thereby improving chlamydial propagation (Oksaharju et al., 2009; Chiba et al., 2015). In addition, eosinophils are essential for tissue repair after genital *C. trachomatis* infection. Since eosinophils are the primary source of IL-4 in the upper genital tract, they are indirectly responsible for the proliferation of endometrial stromal cell. The robust Th2 immunity elicited by *C. trachomatis* infection in the female genital tract may therefore be regulated through IL-4 signaling (Vicetti Miguel et al., 2017), which could, to some extent, explain the role of IL-4-producing eosinophils in preventing the *C. trachomatis*-induced upper genital tract damages. Furthermore, there have been studies documenting the infection of neutrophils with *Chlamydia*. Neutrophils have a very short lifespan compared to other immune cells and exist for a mere 5 h prior to undergoing spontaneous apoptosis. To this end, researchers have shown great interest in establishing how *Chlamydia* persist in such short-lived cells. In the primary human neutrophil infection model, Arup and other groups (Frazer et al., 2011; Sarkar et al., 2015) have demonstrated that the chlamydial infection of neutrophils delays apoptosis in these cells and prolongs their longevity, by activating both ERK1/2 and PI3K/Akt survival signaling pathways. Intriguingly, Nuria and colleagues (Rodriguez et al., 2005) found that polymorph-nuclear neutrophils infected with *C. pneumoniae* could amplify chlamydial replication in epithelial cells *in vivo* through MYD88-dependent signaling. Infection of epithelial cells with a plasmid-bearing *C. trachomatis* released a soluble factor (known as granulocyte-macrophage colony-stimulating factor) that provoked the activation of neutrophils and enhanced their survival (Lehr et al., 2018). In addition, *C. trachomatis* CPAF was shown to target formyl peptide receptor 2 and lead to neutrophil dysfunction by preventing several neutrophil defenses, including the signature oxidative burst and the formation of extracellular traps (Rajeeve et al., 2018).

In summary, the interactions between innate immune cells and *Chlamydia* are serovar- and host-cell specific. Furthermore, the *Chlamydia* spp. employ multiple mechanisms to manipulate innate immune responses and ensure their persistence, further contributing to the pathogenesis of chronic chlamydial infections.

## Concluding Remarks

As an ancient evolutionary defense strategy, the innate immune system mediates a pathogen-specific immune response, elicited by chronic chlamydial infections, which are often conducive to bacterial clearance. Of course, this wily organism has also evolved a wide variety of strategies to counteract the host immune response and to establish a favorable intracellular niche for its survival. It is therefore not surprising that *Chlamydia* interferes with multiple principal signaling pathways that participate in immune recognition, inflammation, apoptosis, and autophagy. Further technological advances are still urgently needed to address the following questions: (1) how the dual roles of the implicated signaling pathways (cytokine production, for instance, serves to either facilitate infection progression or reduce its intensity) determine the final outcome of chlamydial infection; (2) how to balance the Th1/Th2 immune system *via* the regulation of immune modulators; and (3) what is the function of the crucial components produced during the innate immune regulation process. Elucidating these cellular and molecular details may help us delineate the immune evasion mechanisms employed by *Chlamydia*. Future research would benefit from the use of sophisticated animal models and clinical samples, in addition to the immortalized cell lines that have been relied upon to date, in order to characterize chlamydial disease at a systemic level.

## Author Contributions

HC collected literatures and wrote the manuscript. All authors contributed to the drafting of this review.

### Conflict of Interest Statement

The authors declare that the research was conducted in the absence of any commercial or financial relationships that could be construed as a potential conflict of interest.
